# Detailed histological analysis of a thrombectomy-resistant ischemic stroke thrombus: a case report

**DOI:** 10.1186/s12959-021-00262-1

**Published:** 2021-02-22

**Authors:** Senna Staessens, Olivier François, Linda Desender, Peter Vanacker, Tom Dewaele, Raf Sciot, Karen Vanhoorelbeke, Tommy Andersson, Simon F. De Meyer

**Affiliations:** 1 Laboratory for Thrombosis Research, KU Leuven Campus Kulak Kortrijk, E. Sabbelaan 53, 8500 Kortrijk, Belgium; 2 Department of Medical Imaging, AZ Groeninge, Kortrijk, Belgium; 3 Department of Neurology, AZ Groeninge, Kortrijk, Belgium; 4 Department of Neurology, University Hospitals Antwerp, Antwerp, Belgium; 5 Department of Translational Neuroscience, University of Antwerp, Antwerp, Belgium; 6 Department of Pathology, University Hospitals KU Leuven, Leuven, Belgium; 7 Departments of Neuroradiology, Karolinska University Hospital, and Clinical Neuroscience, Karolinska Institutet, Stockholm, Sweden

**Keywords:** Ischemic stroke, Thrombectomy, Thrombus composition, Histology, von Willebrand factor, NETs

## Abstract

**Background:**

Mechanical removal of a thrombus by thrombectomy can be quite challenging. For reasons that are not fully understood, some thrombi require multiple passes to achieve successful recanalization, whereas other thrombi are efficiently removed in a single pass. Since first pass success is associated with better clinical outcome, it is important to better understand the nature of thrombectomy resistant thrombi. The aim of this study was therefore to characterize the cellular and molecular composition of a thrombus that was very hard to retrieve via mechanical thrombectomy.

**Case presentation:**

In a patient that was admitted with a right middle cerebral artery M1-occlusion, 11 attempts using various thrombectomy devices and techniques were required for removal of the thrombus. This peculiar case provided a rare opportunity to perform an in-depth histopathological study of a difficult to retrieve thrombus. Thrombus material was histologically analyzed using hematoxylin and eosin, Martius Scarlet Blue stain (red blood cells and fibrin), Feulgen stain (DNA), von Kossa stain (calcifications) and immunohistochemical analysis of von Willebrand factor, platelets, leukocytes and neutrophil extracellular traps. Histological analysis revealed abnormally high amounts of extracellular DNA, leukocytes, von Willebrand factor and calcifications. Extracellular DNA stained positive for markers of leukocytes and NETs, suggesting that a significant portion of DNA is derived from neutrophil extracellular traps.

**Conclusion:**

In this unique case of a nearly thrombectomy-resistant stroke thrombus, our study showed an atypical composition compared to the common structural features found in ischemic stroke thrombi. The core of the retrieved thrombus consisted of extracellular DNA that colocalized with von Willebrand factor and microcalcifications. These results support the hypothesis that von Willebrand factor, neutrophil extracellular traps and microcalcifications contribute to mechanical thrombectomy resistance. Such information is important to identify novel targets in order to optimize technical treatment protocols and techniques to increase first pass success rates.

## Introduction

Acute ischemic stroke is in the majority of cases caused by a thromboembolic occlusion of the cerebral arteries. Since 2015, several positive thrombectomy trials have dramatically changed the treatment of large vessel occlusions by implementing endovascular procedures as a novel therapeutic strategy [1–5]. One of the major obstacles in this rapidly developing field is the fact that thrombi tend to differ in consistency and removability. Indeed, 60–75% of thrombectomy procedures require multiple passes to achieve a Thrombolysis in Cerebral Infarction (TICI) 2b-3 recanalization rate [6, 7]. Moreover, 10–20% of thrombi cannot be removed from the cerebral circulation [8].

Most importantly, first-pass complete reperfusion (TICI 3) is an independent factor for favorable outcome and this first-pass effect has become an important aim in mechanical thrombectomy [6, 7, 9]. Information as to why certain thrombi are difficult to retrieve is currently largely lacking but beside thrombus location, thrombus size and vascular access, thrombus composition is believed to play a key role in removability [9].

In this study, we investigated in detail the molecular and cellular composition of a nearly-irretrievable thrombus and report an atypical histological composition of this thrombectomy-resistant thrombus.

## Case presentation

### Clinical and procedural information

A 63-year old female was admitted in the late evening to a primary stroke center at a peripheral hospital with an acute left sensorimotor hemi-syndrome and visual extinction, during the same day she had noticed some transient hemianopia. An initial non-contrast-enhanced computed tomography (NCCT) revealed no intracerebral hematoma or other abnormalities but as the exact time of symptom onset was unknown, no intravenous thrombolysis was administered. The patient was treated with aspirin and low-molecular heparin, after which the symptoms disappeared spontaneously overnight.

**Fig. 1:**
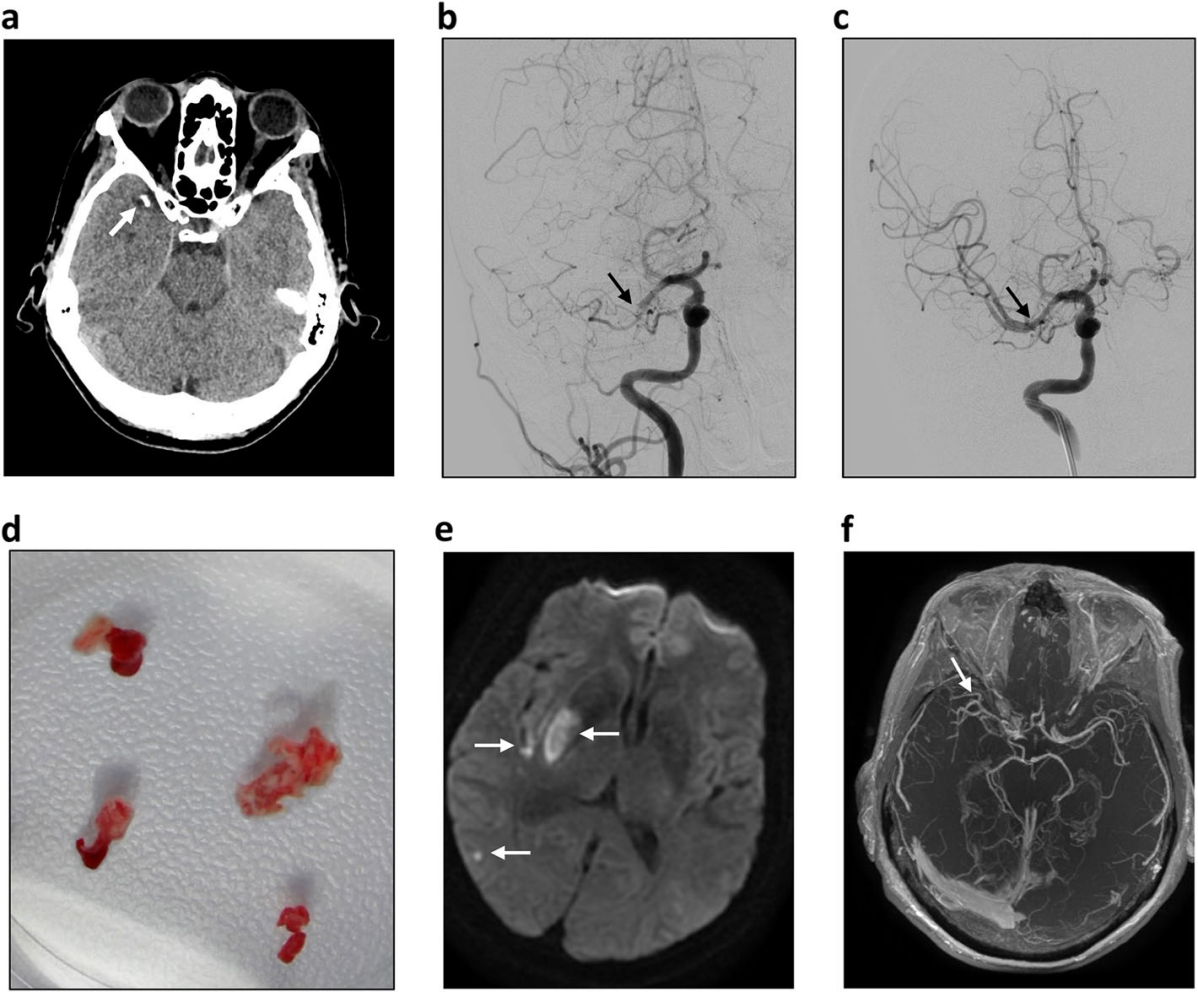
Radiological imaging of the patient. A non-contrast CT scan of the head revealed a right hyperdense middle cerebral artery sign (average 138 HU, white arrow), with already infarction in the basal ganglia (not shown) in the right hemisphere but no signs of intracranial hemorrhage (**a**). On DSA, initial runs confirmed a complete occlusion of the distal M1-segment of the right middle cerebral artery (black arrow) with good pial collateralization (**b**). Final angiographic result after the 11th thrombectomy attempt showing a complete recanalization of the right middle cerebral artery (**c**, black arrow). The thrombus appeared hard and white macroscopically (**d**). DWI-MRI demonstrated restricted diffusion in the right basal ganglia and several smaller cortical and subcortical lesions 24 h after the endovascular treatment (**e**, white arrows) and MRA showed recanalization of the previously occluded segment of the right middle cerebral artery (**f**, white arrow)

Early next morning the patient experienced an acute recurrence of the left sensorimotor hemi-syndrome and repeated imaging with NCCT showed a hyperdense right middle cerebral artery (MCA) sign (average 138 HU), with a partial infarction of the basal ganglia and no signs of intracranial hemorrhage (Fig. 1a). CT-angiography confirmed distal M1 and inferior branch M2 right-sided MCA occlusions with good pial collateralization (Fig. 1b).

The patient was transferred to AZ Groeninge Hospital in Kortrijk, Belgium, for endovascular therapy, arriving 2.5 h after symptom onset. At arrival, the patient had a score of 11 at the National Institute of Health Stroke Scale (NIHSS). Her medical history included a successful treatment in 2001 of an invasive, hormone-receptor positive ductal breast carcinoma but no cardiovascular risk factors except a minor dyslipidemia, for which she was treated with rosuvastatin.

After a brief discussion in the multidisciplinary stroke team, an endovascular procedure was performed under general anesthesia. Vascular access was achieved 25 min after arrival with insertion of an 80 cm long 8French (F) femoral arterial sheath (Arrow, Morrisville, US) into the right common carotid artery. A 95 cm 8F balloon guide catheter (BGC; FlowGate, Stryker Neurovascular, Fremont, California, USA) was inserted through the sheath and positioned with the tip in the distal cervical segment of the internal carotid artery. A 0.021-in. microcatheter (Prowler Select Plus, Codman Neurovascular J&J, Raynham, Massachusetts, US) was advanced over a 0.014″ microwire (Transend-EX Platinum, Stryker Neurovascular, Fremont, California, USA) through the occluded distal M1- and inferior M2-segments of the right MCA. The microwire was exchanged for a 5 × 33 mm stent retriever thrombectomy device (EmboTrap II, Cerenovus – J&J, Wokingham, UK), which was subsequently deployed in the occluded parts of the M1-M2 segments. Under temporary BGC-occlusion but without an intermediate aspiration catheter, the stent retriever was slowly retracted in three consecutive unsuccessful attempts to retrieve the clot. Technically identical procedures where then performed another three times but with a 4 × 30 mm Trevo-XP Provue stent retriever (Stryker Neurovascular, Fremont, California, US). These retrieval attempts were also unsuccessful with an unchanged complete occlusion. The initial stent retriever strategy was then changed to direct contact aspiration utilizing a 125 cm 5F Sofia intermediate catheter (MicroVention, Aliso Viejo, California, US), inserted through the BGC over the microcatheter and microwire, reaching the occluded segment of the right MCA. Three attempts with manual aspiration were made with a 20 cc syringe, but the occlusion remained unchanged with no clot removal. Finally, the Sofia intermediate catheter was replaced by the slightly larger Catalyst-6 (Stryker Neurovascular, Fremont, California, US) aspiration catheter and another two attempts were made with a combination PROTECT^PLUS^ technique [10] in conjunction with the EmboTrap stent retriever. At the second of these two attempts, focusing on holding the clot by a steady negative pressure in the aspiration catheter during withdrawal of the stent retriever, microcatheter and aspiration catheter simultaneously under BGC-induced carotid flow-arrest, a successful retrieval of a complete clot was achieved in the eleventh removal attempt, resulting in a TICI 2c revascularization (Fig. 1c). Macroscopically, the thrombus appeared unusually hard with a clear white coloring (Fig. 1d).

The day after the endovascular treatment a brain magnetic resonance imaging (MRI) investigation was performed including diffusion-weighted- (DWI), fluid-attenuated inversion recovery (FLAIR) and contrast enhanced T1-weighted images. DWI demonstrated restricted diffusion in the right basal ganglia and several small cortical and subcortical lesions (Fig. 1e), consistent with acute small embolic infarctions. Magnetic Resonance Angiography (MRA-TOF) showed a complete recanalization of all branches of the right middle cerebral artery (Fig. 1f).

A screening was made with an implantable loop recorder in collaboration with the Cardiology Department to search for an underlying cardioembolic source and the patient was thoroughly investigated regarding a potential atherothrombotic source or an underlying coagulopathy, but no causative pathology could be found.

### Thrombus histology

**Fig. 2:**
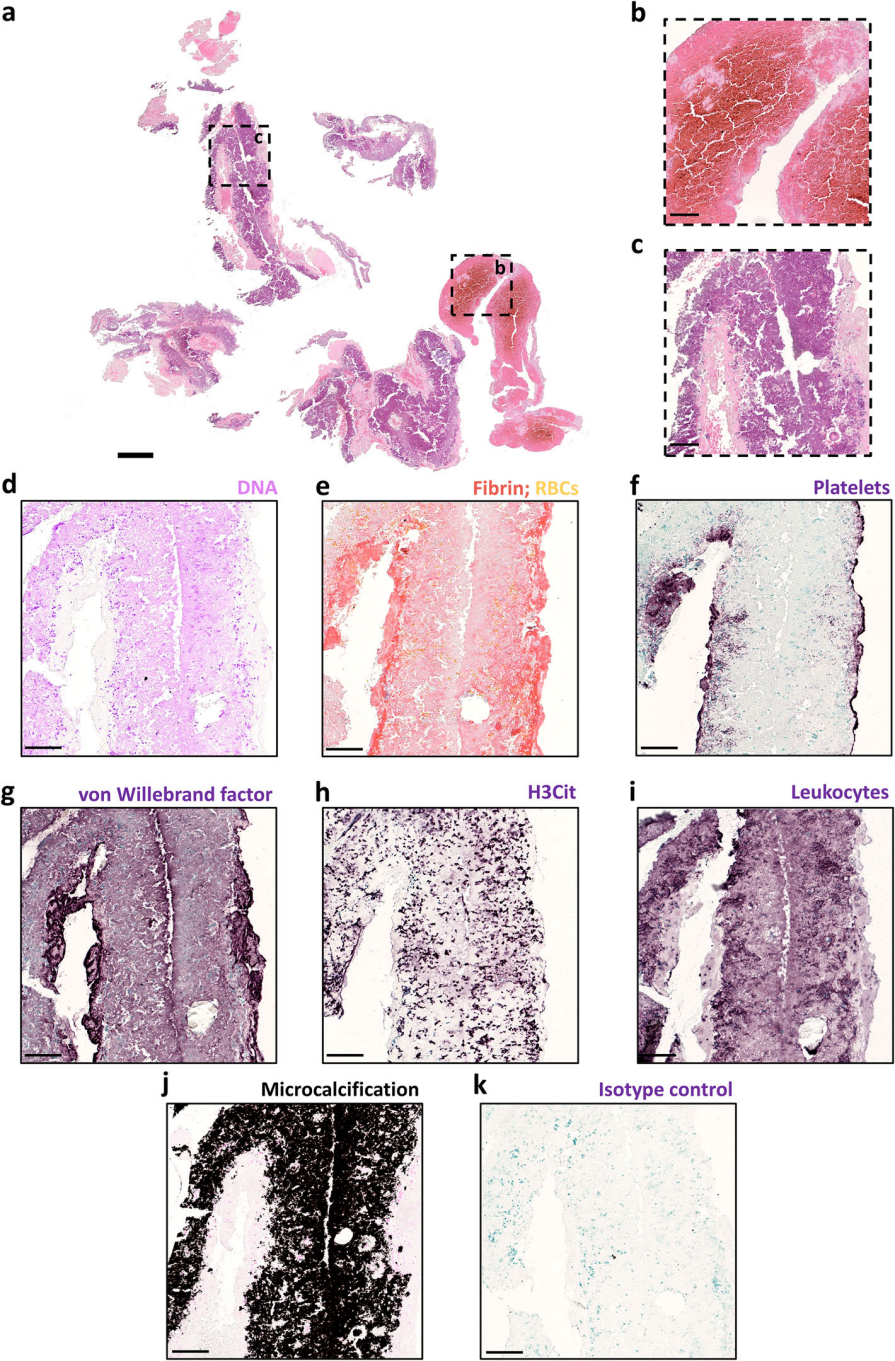
Histological analysis. The thrombus was stained for H&E, MSB, DNA, platelets, VWF, NETs (H3Cit), leukocytes and microcalcifications. H&E staining (**a**) was used to visualize the overall thrombus composition and organization, indicating the presence of an RBC-rich area in red (**b**) and a large DNA-rich area in purple (**c**). Feulgen staining, a specific DNA stain, confirmed the presence of extracellular DNA with few intact nuclei in this area (**d**, pink). MSB staining and the platelets staining revealed the presence of fibrin (**e**, red) and platelets (**f**, purple) on the surface of the thrombus, whereas VWF (G, purple) was present throughout the DNA-rich area. H3Cit staining (purple) showed the presence of extracellular H3Cit positive smears (**h**, purple) and leukocyte staining showed a large smear with little to no intact leukocytes present (**i**, purple), indicating that extracellular DNA originated from leukocytes and to some extent from neutrophils. Von Kossa staining showed abundant microcalcifications (**j**, black). A representative image of an isotype control for the VWF staining is shown in panel K. Scale bars are 500 μm for A and 125 μm for B-K

After thrombus removal, the thrombus was fixed in 4% paraformaldehyde, embedded in paraffin and sectioned in 5 μm sections. To obtain a general overview of the thrombus structure, a hematoxylin and eosin (H&E) staining was performed (Fig. 2a-c). H&E analysis showed various regions that included fibrin/platelet aggregations (pink), red blood cells (RBCs; red) and nuclear material (dark blue) (Fig. 2a). Whereas one fragment was more RBC-rich (Fig. 2b; red), the majority of the thrombus stained largely dark blue or purple, indicative of nucleic acid material with little RBCs (Fig. 2c; blue smears). To confirm the presence of extracellular DNA, a highly specific DNA stain (Feulgen’s reaction) was performed. High amounts of extensive DNA networks were found throughout the majority of the thrombus (Fig. 2d, pink). To specifically identify fibrin and RBCs, a Martius Scarlet Blue (MSB) staining was performed, which confirmed the absence of RBCs in the DNA-rich thrombus parts (Fig. 2e). Fibrin (red on MSB staining) was predominantly found on the outer rim of the thrombus but not in the DNA-rich inner core (Fig. 2e). Similarly, immunohistochemical platelet staining showed the presence of platelets only on the outer shell of the thrombus (Fig. 2f; purple). We previously identified von Willebrand factor (VWF) as an important constituent of stroke thrombi, conferring resistance to fibrinolytic therapy in mice [11]. Strikingly, using an immunohistochemical VWF staining, very high amounts of von Willebrand factor-positive smears were identified in the DNA-rich area (Fig. 2g, purple).

Given our recent findings showing the presence of neutrophil extracellular DNA traps (NETs) in stroke thrombi, we stained for citrullinated histone H3 (H3Cit), a marker of NETosis [12]. H3Cit-positive staining was found within nuclei and also extracellularly as smears (Fig. 2h, purple). Immunostaining for the leukocyte marker CD45 showed an abundant and dense signal in the DNA-rich areas (Fig. 2i, purple). Together, the staining results for DNA, H3Cit and CD45 suggest that leukocytes could be the origin of extracellular DNA, of which a significant portion is decondensed, as seen in NETs. The uncommon structure is further underlined by the von Kossa staining, which revealed the exceptional presence of high amounts of microcalcifications (Fig. 2j, black).

## Discussion

In patients, fast removal of the thrombus, preferably via a first successful pass, is desirable for good clinical outcome. However, not all thrombi are easily removable. It has become clear that the composition of the thrombus is an important determinant of thrombus removability. RBC-rich thrombi are more easily retrieved in comparison to more complex fibrin/platelet-rich thrombi [13]. In 10–20% of patients, however, the thrombus cannot be removed, despite multiple attempts. The composition of difficult to retrieve thrombi remains unclear. In this case report, we describe the atypical structure of a nearly thrombectomy-resistant thrombus. We found that more than half of the thrombus is composed of extracellular DNA, which is most likely originating from leukocytes and NETs. Interestingly, the presence of extracellular DNA is associated with unusually large amounts of VWF and microcalcifications.

Recently, we described the typical structure of RBC-rich and platelet-rich areas commonly found in ischemic stroke thrombi [14]. In particular, platelet-rich thrombus material was characterized by the presence of extracellular DNA and VWF, which were rather absent in RBC-rich areas [14]. Based on our own results and on data from literature, this thrombectomy-resistant thrombus is atypical in three ways. First, the amounts of extracellular DNA and VWF are exceptionally high compared to what is typically found in stroke thrombi. Second, the presence of extracellular DNA and VWF is not associated with platelet-rich thrombus material, as typically seen in other thrombi. Third, both VWF and extracellular DNA colocalized with a significant amount of microcalcifications. Such abundant microcalcification is very rarely observed in our larger collection of stroke thrombi (unpublished observation). Possibly, this atypical composition, explains the high resistance against thrombectomy. Thrombus composition is known to influence the coefficient of friction and the integration of the thrombus within the thrombectomy device [15, 16]. Longstaff et al. showed that extracellular DNA, in particular NETs, increase the mechanical resistance of thrombi [17]. In addition, both Ducroux et al. and Novotny et al. found a correlation between the amount of NETs and the amount of thrombectomy passes required to remove the thrombus [18, 19]. Besides NETs, calcifications have also been shown to negatively impact thrombectomy success rates [20]. In fact, calcified thrombi are associated with increased thrombus stiffness [21]. Possibly, the struts of thrombectomy devices might not be able to integrate in the thrombus due to the presence of NETs and/or microcalcifications.

The uncommon structure of this thrombus raises the intriguing question about its origin or underlying pathology. Our findings suggest an interplay between VWF, NETs and calcification. Interestingly, high VWF antigen levels are associated with aortic arch and carotid artery calcification in ischemic stroke patients [22]. Coscas et al. have recently shown that cell-free DNA is able to induce precipitation of calcium phosphate apatite crystals in the arterial wall [23]. In addition, VWF can bind to both histones and DNA [24–26]. Together with the high leukocyte-positive signal, it is tempting to speculate that the retrieved material in this study might be part of a ruptured atherosclerotic plaque. Unfortunately, due to the nature of the thrombectomy procedure, it is not possible to know to original orientation of the retrieved thrombus in the blood vessel. A full clinical work-up of the patient could, however, not resolve the underlying stroke etiology, leading to the classification of an embolic stroke of undetermined source.

In conclusion, this case-report describes a unique case in which we were able to study the composition of a stroke thrombus that was thrombectomy-resistant. Interestingly, our study showed an atypical structure compared to the common structural features found in ischemic stroke thrombi. The bulk of the retrieved thrombus consisted of extracellular DNA that colocalized with VWF and microcalcifications, which together could possibly explain the resistance to mechanical removal. Such information can help the development of improved protocols and technologies that increase first pass success rates, ultimately leading to better clinical outcome of thrombectomy-treated patients.

## Data Availability

The datasets used and/or analyzed during the current study are available from the corresponding author on reasonable request.

## Abbreviations

TICI: Thrombolysis in cerebral infarction scale
NIHSS: National institute of health stroke scale
BGC: Balloon guide catheter
CT: Computed tomography
CTA: Computed tomography angiography
MRI: Magnetic resonance imaging
DWI: Diffusion weighted imaging
FLAIR: Fluid-attenuated inversion recovery
MRA-TOF: Magnetic resonance angiography
H&E: Hematoxylin and eosin
RBCs: Red blood cells
MSB: Martius scarlet blue
VWF: Von willebrand factor
NETs: Neutrophil extracellular traps
H3Cit: Citrullinated histone H3
CD45: Cluster of differentiation 45
